# Supplementary material for: Comparative genomics provides new insights into the diversity, physiology, and sexuality of the only industrially exploited tremellomycete: Phaffia rhodozyma
Source: BMC Genomics. 2016 Nov 9;17:901. doi: 10.1186/s12864-016-3244-7 (PMC5103461; doi:10.1186/s12864-016-3244-7)
Supplement: Additional file 6: — List of orphan genes with links to PFAM (related to Additional file 1: Table S1). (ZIP 1428 kb) [file 12864_2016_3244_MOESM6_ESM.zip › BLAST_HTML_FTR/G01200_P.html]

BLAST Search Results


```
BLASTP 2.2.27+


Reference:
Stephen F. Altschul, Thomas L. Madden, Alejandro A. Schäffer,
Jinghui Zhang, Zheng Zhang, Webb Miller, and David J. Lipman (1997),
"Gapped BLAST and PSI-BLAST: a new generation of protein database
search programs", Nucleic Acids Res. 25:3389-3402.


Reference for
composition-based statistics:
Alejandro A. Schäffer, L. Aravind, Thomas L. Madden, Sergei
Shavirin, John L. Spouge, Yuri I. Wolf, Eugene V. Koonin, and
Stephen F. Altschul (2001), "Improving the accuracy of PSI-BLAST
protein database searches with composition-based statistics and
other refinements", Nucleic Acids Res. 29:2994-3005.


Database: nr
           71,551,133 sequences; 26,053,659,533 total letters


Query= G01200_P

Length=188
                                                                      Score     E
Sequences producing significant alignments:                          (Bits)  Value

emb|CED82902.1|  AT hook-like [Xanthophyllomyces dendrorhous]          370    7e-128
dbj|GAC72981.1|  amino acid transporters [Pseudozyma antarctica T...  40.0    0.86  
gb|KKK23926.1|  GPI-anchor biosynthetic protein [Aspergillus ochr...  40.4    0.94  
gb|KKK21906.1|  hypothetical protein ARAM_004737 [Aspergillus ram...  40.0    1.1   
gb|AAG00239.1|AC002130_4  F1N21.6 [Arabidopsis thaliana]              38.5    3.3   


 >emb|CED82902.1| AT hook-like [Xanthophyllomyces dendrorhous]
Length=208

 Score =  370 bits (951),  Expect = 7e-128, Method: Compositional matrix adjust.
 Identities = 187/187 (100%), Positives = 187/187 (100%), Gaps = 0/187 (0%)

Query  1    MSKLLEQQAIDRANGIDTPLPELKPILPPGPKRGRGRPPGVKNGEGQKHQKNNLMNNVPP  60
            MSKLLEQQAIDRANGIDTPLPELKPILPPGPKRGRGRPPGVKNGEGQKHQKNNLMNNVPP
Sbjct  22   MSKLLEQQAIDRANGIDTPLPELKPILPPGPKRGRGRPPGVKNGEGQKHQKNNLMNNVPP  81

Query  61   NPLAPKSTTTYPKGPGSTGTGKRGRPPGVKNGQGKNTAIKKARSSEEDGQGEIVEEDGQG  120
            NPLAPKSTTTYPKGPGSTGTGKRGRPPGVKNGQGKNTAIKKARSSEEDGQGEIVEEDGQG
Sbjct  82   NPLAPKSTTTYPKGPGSTGTGKRGRPPGVKNGQGKNTAIKKARSSEEDGQGEIVEEDGQG  141

Query  121  EIVEEDDEEDAYGIQSSSPNGQKSAKDAAAMKKGGHPYMPAYISAPMTTHSTVIPPTGAK  180
            EIVEEDDEEDAYGIQSSSPNGQKSAKDAAAMKKGGHPYMPAYISAPMTTHSTVIPPTGAK
Sbjct  142  EIVEEDDEEDAYGIQSSSPNGQKSAKDAAAMKKGGHPYMPAYISAPMTTHSTVIPPTGAK  201

Query  181  RGRPPKK  187
            RGRPPKK
Sbjct  202  RGRPPKK  208


>dbj|GAC72981.1| amino acid transporters [Pseudozyma antarctica T-34]
Length=558

 Score = 40.0 bits (92),  Expect = 0.86, Method: Compositional matrix adjust.
 Identities = 39/128 (30%), Positives = 56/128 (44%), Gaps = 17/128 (13%)

Query  17   DTPLPELKPILPPGPKRGRGRPPGVKNGEGQKHQKNNLMNNVPPNPLA------PKSTTT  70
            DT +       P  P R RGRPP  K+         ++  +  P   A      PKS +T
Sbjct  192  DTSIDMSAAATPNAPARKRGRPP--KSAYAAVDASVSMPTDATPEAPARKRGRPPKSAST  249

Query  71   YPKGPGSTGT---GKRGRPPGVKNGQGKN-----TAIKKARSSEEDGQGEIVEED-GQGE  121
             P    ST T    +RGRPPG  + +         A++++RS E+ G+ ++   D G  E
Sbjct  250  APNTSVSTSTPAAKRRGRPPGSSSAKKDARTAAPDALRRSRSVEDSGESDVSLVDGGSDE  309

Query  122  IVEEDDEE  129
              E DD E
Sbjct  310  ASEADDAE  317


>gb|KKK23926.1| GPI-anchor biosynthetic protein [Aspergillus ochraceoroseus]
Length=1545

 Score = 40.4 bits (93),  Expect = 0.94, Method: Composition-based stats.
 Identities = 39/164 (24%), Positives = 60/164 (37%), Gaps = 25/164 (15%)

Query  26   ILPPGPKRGRGRPPGVKNGEGQKHQKNNLMNNVPPNPLAPKSTTTYPKGP--GSTGTGKR  83
            ++   P +G       K   G+   K+  M   PP  +A     T P+     +  T +R
Sbjct  21   VMQAAPDQGENNERPTKKARGRPRSKSAEMK--PPVHIATDVAATAPQSSDTATKRTTRR  78

Query  84   GRPPGVKNGQGKNTAIKKARSSEEDGQGEIVEEDGQGEIVEEDDEEDAYGIQSSSPNGQK  143
            GRP G +N                 GQ   +E +GQ    +E D +D  G    +P  +K
Sbjct  79   GRPKGSRN----------------SGQ---MEAEGQDITPQETDGDDGIGALQGAPQPKK  119

Query  144  SAKDAAAMKKGGHPYMPAYISAPMTTHSTVIPPTGAKRGRPPKK  187
            S K A    +G     P+      T       PT  +  +P +K
Sbjct  120  STKAAKGATRGRRK--PSAEKQIKTDGEFEYTPTSTRHKKPTEK  161


>gb|KKK21906.1| hypothetical protein ARAM_004737 [Aspergillus rambellii]
Length=1601

 Score = 40.0 bits (92),  Expect = 1.1, Method: Composition-based stats.
 Identities = 39/164 (24%), Positives = 60/164 (37%), Gaps = 25/164 (15%)

Query  26   ILPPGPKRGRGRPPGVKNGEGQKHQKNNLMNNVPPNPLAPKSTTTYPKGP--GSTGTGKR  83
            ++   P +G       K   G+   K+  M   PP  +A     T P+     +  T +R
Sbjct  21   VMQAAPDQGENNERPTKKARGRPRSKSAEMK--PPVHIATDVAATAPQSSDTATKRTTRR  78

Query  84   GRPPGVKNGQGKNTAIKKARSSEEDGQGEIVEEDGQGEIVEEDDEEDAYGIQSSSPNGQK  143
            GRP G +N                 GQ   +E +GQ    +E D +D  G    +P  +K
Sbjct  79   GRPKGSRN----------------SGQ---MEAEGQDITPQETDGDDGIGALQGAPQPKK  119

Query  144  SAKDAAAMKKGGHPYMPAYISAPMTTHSTVIPPTGAKRGRPPKK  187
            S K A    +G     P+      T       PT  +  +P +K
Sbjct  120  STKAAKGATRGRRK--PSAEKQIKTDGEFEYTPTSTRHKKPTEK  161


>gb|AAG00239.1|AC002130_4 F1N21.6 [Arabidopsis thaliana]
Length=901

 Score = 38.5 bits (88),  Expect = 3.3, Method: Composition-based stats.
 Identities = 26/68 (38%), Positives = 34/68 (50%), Gaps = 6/68 (9%)

Query  30   GPKRGRGRPPGVKNGEGQKHQKNNLMNNVPPNPLA-PKSTTTYPKGPG--STGTGK-RGR  85
             P+R R  P   ++     H   +  N  PP  L+ P     +   P   S+GTG+ RGR
Sbjct  809  APRRWRNAPNVAQSQYSNPHVAQSQSN--PPIALSQPTQIPQHSSAPAAPSSGTGRARGR  866

Query  86   PPGVKNGQ  93
            PPGVKNGQ
Sbjct  867  PPGVKNGQ  874


Lambda      K        H        a         alpha
   0.308    0.132    0.388    0.792     4.96 

Gapped
Lambda      K        H        a         alpha    sigma
   0.267   0.0410    0.140     1.90     42.6     43.6 

Effective search space used: 750352639660


  Database: nr
    Posted date:  Sep 23, 2015 12:05 AM
  Number of letters in database: 26,053,659,533
  Number of sequences in database:  71,551,133


Matrix: BLOSUM62
Gap Penalties: Existence: 11, Extension: 1
Neighboring words threshold: 11
Window for multiple hits: 40
```
